# miRNAs Involved in M1/M2 Hyperpolarization Are Clustered and Coordinately Expressed in Alcoholic Hepatitis

**DOI:** 10.3389/fimmu.2019.01295

**Published:** 2019-06-07

**Authors:** Adam Kim, Paramananda Saikia, Laura E. Nagy

**Affiliations:** Department of Inflammation and Immunity, Northern Ohio Alcohol Center, Center for Liver Disease Research, Cleveland Clinic, Lerner Research Institute, Cleveland, OH, United States

**Keywords:** alcoholic hepatitis, alcoholic liver disease, kupffer cells, macrophage, monocyte, peripheral blood mononuclear cells, miRNA expression, small RNA sequencing

## Abstract

The innate immune system, including monocytes/macrophages, is critical to the progression of alcoholic liver disease (ALD). In response to chronic ethanol, Kupffer cells, the resident macrophage of livers, and peripheral monocytes become sensitized to bacterial lipopolysaccharides (LPS), express more pro-inflammatory cytokines and exhibit macrophage M1/M2 hyperpolarization. Since miRNAs play an important role in the regulation of M1/M2 polarization, we hypothesized that miRNAs regulating macrophage polarization would be dysregulated after chronic ethanol consumption. miRNA sequencing data from Kupffer cells isolated from rats fed an ethanol diet vs. control diet and qPCR data from PBMCs isolated from alcoholic hepatitis (AH) patients and healthy controls were used to assess the role of miRNAs in macrophage hyperpolarization in ALD. Differential expression analyses revealed 40 misregulated miRNAs in Kupffer cells from the chronic ethanol-fed rats compared to pair-fed controls. Nine of these miRNAs are known to be associated with macrophage polarization and consist of a mixture of M1- and M2-associated miRNAs, indicative of hyperpolarization. Twenty-three of the 40 differentially expressed miRNAs were localized to miRNA clusters throughout the genome. Correlation analyses revealed that miRNAs in three of these clusters were co-regulated and located within antisense non-coding RNAs. Similar to Kupffer cells from ethanol-fed rats, M1 and M2 polarization markers, as well as sensitivity to LPS, were elevated in PBMCs from AH patients compared to healthy controls. These increases were associated with an up-regulation of polarization-associated miRNAs, including miR-125a-5p, a miRNA associated with hyperpolarization. miR-125a-5p is clustered in the genome with other miRNAs inside a host gene, Spaca6, which was also upregulated in PBMCs, as well as isolated monocytes, from AH patients. Finally, correlation analyses revealed co-regulation of human polarization-associated miRNA clusters. While expression of polarization-associated miRNAs in clusters was upregulated in AH compared to healthy controls, co-regulation of the miRNAs within a cluster was independent of disease state. Together, these results reveal that global changes in miRNA regulation are associated with polarization phenotypes in Kupffer cells from rat after chronic ethanol as well as in PBMCs from patients with AH. Importantly, polarization-associated miRNAs were localized to coordinately regulated clusters.

## Introduction

Alcohol-related liver disease (ALD) is the leading cause of liver-related disorders and contributes to 25% of alcohol-related deaths across the world (1, 2). ALD is characterized by progressive liver steatosis, fibrosis, and cirrhosis; alcoholic hepatitis (AH), an acute inflammatory condition, can occur at any point along the disease spectrum and, in severe AH, has a high rate of short-term mortality (3). While the causes of AH are unknown, consumption of alcohol increases gut permeability, leading to translocation of both microbes and microbial products, including lipopolysaccharide (LPS), β-glucan and other pathogen associated molecular patterns (PAMPs), into the portal circulation, activating innate immune responses in the liver (4, 5).

The first immune cells to respond to these PAMPs are Kupffer cells, the resident macrophage in the liver (6, 7). Chronic ethanol exposure sensitizes Kupffer cells to activation by PAMPs, leading to exacerbated pro-inflammatory responses (6–8). Pro- and anti-inflammatory responses of macrophages can be influenced by their polarization state (9). Macrophage polarization is a spectrum of activation states, though little is known as to how these differing phenotypes function *in vivo* and at an individual cell level. *In vitro* studies of polarization focus on the phenotypes generated by exposure to specific activating signals, such as LPS/IFNγ-induced M1 (pro-inflammatory) polarization and IL-10 or IL-4 induction of specific types of M2 (anti-inflammatory) polarized macrophages. Polarization is typically characterized by expression of cell surface markers that elicit different pro- and anti-inflammatory effects, including CD86 (M1) and CD206 (M2) (10, 11).

The polarization phenotype of Kupffer cells in response to ethanol is complex, with increased expression of both M1 and M2 polarization markers, as well as increased expression of both pro- and anti-inflammatory mediators. This complex response has been termed hyperpolarization. Chronic ethanol feeding to mice increased expression of M1 and M2 markers in the liver, indicative of a hyperpolarized state (12). Kupffer cells isolated from rats fed a chronic ethanol diet were predominantly M1 compared to pair-fed controls (13). Interestingly, the ethanol-induced M1 polarization is modifiable; M1 macrophages could be shifted to an M2 phenotype, either when anti-inflammatory cannabinoid signaling was stimulated (12) or in response to treatment with adipose-derived adiponectin (13). Very little is known about the mechanisms for hyperpolarization and the impact of these complex phenotypes on the development of chronic ethanol-induced inflammation in the liver (12–15). In humans, resident macrophages in liver biopsies from AH patients are also hyperpolarized with increased expression of M1 and M2 markers (15). While functional studies have not been performed on human Kupffer cells, peripheral blood mononuclear cells (PBMCs) isolated from AH patients have been used as a surrogate to model the pro-inflammatory nature of the innate immune system (8). Similar to the rodent Kupffer cells, PBMCs isolated from AH patients are also hypersensitive to LPS and respond with increased pro-inflammatory cytokine expression, implicating a possible role for increased M1 polarization in peripheral immune cells as well as Kupffer cells. Additionally, fluorescence microscopy of PBMCs isolated from patients with alcohol use disorders but without any underlying pathology had increased CD163 (M2b) expression in CD14+ monocytes (16). Acute alcohol consumption also increases expression of CD86, CD68, CD163, and CD206 among CD14+ monocytes (17). These data together implicate a complex role for macrophage polarization after alcohol consumption.

miRNAs are small non-coding RNAs that regulate gene expression by binding to mRNAs and inhibiting translation. They are transcribed as pri-miRNAs via 2 mechanisms: as (1) solitary transcriptional units or (2) co-transcribed and processed from the intron of host mRNAs. pri-miRNAs are then processed into two mature miRNAs, −5p and −3p (18, 19). Small RNA-sequencing and microarrays from M1 and M2 polarized macrophages, as well as non-polarized monocytes, have revealed dynamic regulation of polarization by dozens of miRNAs (20). Recently, work by our group and others found miRNAs to play important regulatory roles in response to ethanol (17, 21–24). miR-181b-3p was down regulated in Kupffer cells isolated from chronic ethanol-fed rats, which leads to upregulation of importin α5 and NFκB signaling. miR-155 is expressed in Kupffer cells isolated from mice fed an ethanol diet, and loss of miR-155 attenuates the response to LPS (23–25). miR-27a is induced by acute alcohol consumption in humans and shifts macrophages toward M2 polarization (17).

While previous studies have detected roles for individual miRNAs in disease, few have investigated the global regulation of miRNAs and how miRNAs may collectively function to change cellular phenotypes. Using two models of innate immune dysfunction, Kupffer cells from chronic ethanol-fed rats and PBMCs isolated from AH patients, we hypothesized miRNAs associated with polarization might be dysregulated. Using our previously published small RNA sequencing data (21), we sought to understand how miRNAs are globally regulated in response to ethanol in rats. Among differentially expressed miRNAs, we found enrichment of miRNAs associated with M1/M2 polarization in Kupffer cells from rats chronically-fed an ethanol diet. Many of these miRNAs were clustered throughout the genome. Interestingly, some of the polarization-associated miRNAs were found in introns of regulatory host genes; co-expression analyses revealed co-regulation of certain miRNA clusters. These clusters contain additional miRNAs that function in immune signaling and polarization, thus increasing the potential for coordinated regulation of immune mediators by miRNAs. Finally, peripheral blood mononuclear cells (PBMCs) isolated from AH patients, as well as purified CD14^+^ and CD16^+^ monocytes, also had dysregulated expression of miRNAs associated with polarization. These data together implicate the regulation of miRNAs as an important aspect of innate immune dysfunction in response to alcohol.

## Materials and Methods

### Small RNA-Sequencing of Kupffer Cells Isolated From Ethanol-Fed Rats

Small RNA-sequencing data from rat Kupffer cells was obtained as described previously (21). Briefly, adult male Wistar rats from Envigo (Indianapolis, IN) were randomly assigned to pair-fed or ethanol-fed groups. Ethanol-fed rats were allowed free access to a liquid Lieber-Decarli high-fat liquid diet (#710260; Dyets, Bethlehem, PA) containing 17% of the calories (3.3% vol/vol) from ethanol for 2 days and then a liquid diet containing 35% of the calories (6.7% vol/vol) from ethanol for 4 weeks. Control rats were pair-fed a liquid diet in which maltose dextrin was substituted isocalorically for ethanol over the entire feeding period. Kupffer cells were isolated and cultured as described previously (26). After 18 h in culture, cells were challenged with 10 ng/mL LPS (Escherichia coli strain 0111:B4, Sigma-Aldrich, St. Louis, MO). Small RNA was isolated using miRNeasy Mini Kit (Qiagen, Germantown, MD) and sequenced following the Small RNA Sample Preparation Protocol (Illumina, San Diego, CA). The library was prepared from 10 ng of total RNA per sample according to the manufacturer's instructions (TruSeq, Small RNAKit, Illumina). Single-stranded complementary DNAs were created with SuperScriptII Reverse Transcriptase, and double-stranded complementary DNAs were generated by way of polymerase chain reaction using adapter-specific primers. Purified libraries were sequenced using HiSeq2000 (Illumina).

### Alcoholic Hepatitis and Healthy Control Patient Selection

Enrolled patients had a confirmed diagnosis of alcoholic hepatitis by clinicians at the Cleveland Clinic and University Hospitals, Cleveland, based on medical history, physical examination, and laboratory results, according to the guidelines of the American College of Gastroenterology [https://gi.org/clinical-guidelines/]. Healthy control subjects were recruited from the Clinical Research Unit at the Cleveland Clinic. The study protocol was approved by the Institutional Review Board for the Protection of Human Subjects in Research at the Cleveland Clinic and University Hospitals, Cleveland. All methods were performed in accordance with the IRB's guidelines and regulations and written informed consent was obtained from all subjects.

### Isolation of Human PBMCs

Isolation of PBMCs from human blood was performed by density gradient centrifugation on Ficoll-Paque PLUS. Briefly, 1 mL of freshly collected Buffy Coat was mixed at a ratio of 1:5 (vol/vol) with PBS at 37°C and the mixture was divided and layered onto 8 mL Ficoll-Paque PLUS in two 15 mL conical centrifuge tubes. After centrifugation at 400 × g for 30 min at 20°C (no brake), buffy coat fractions were collected, pooled, resuspended in culture media (RPMI-1640 supplemented with 100 μM Penicillin-streptomycin and 10 vol% FBS) and centrifuged at 400 × g for 15 min at 20°C. The pellets were resuspended in 8 mL of culture media, counted, and again centrifuged at 400 × g for 8 min at 20°C. Cells were then cryo-perserved by resuspending in freezing media (50% culture media, 40% FBS, 10% DMSO) at a concentration of 1.5 × 10^6^ cells/mL, and allowed to freeze slowly to −80°C in a styrofoam container. For long-term preservation, cells were stored in liquid nitrogen. For experiments, cells were thawed at 37 °C, then added slowly to 8 mL of warm culture media. After centrifugation at 400 × g for 8 min at 20°C, cells were resuspended and cultured in 96-well plates in a humidified atmosphere (5% CO_2_, 37°C). After 18 h, cells were washed with media and challenged with 10 ng/ml LPS for another 60 min.

### Isolation of CD14+ and CD16+ Monocytes

CD14+ and CD16+ monocytes were isolated from cryopreserved and thawed PBMCs using the EasySep Human Monocyte Enrichment Kit without CD16 depletion (StemCell Technologies, Cambridge, MA) following manufacturer's instructions with a DNAse treatment to prevent cell clumping. After isolation, cells were cultured in tubes and incubated for 24 h prior to RNA extraction.

### Sequencing Analysis, PCA, and Calculation of Differential Expression

Sequencing data was aligned to the rat genome (Rnor_6.0) using Bowtie2 with the –*very-sensitive-local* setting (27). Expression of annotated miRNAs was determined using custom perl scripts. PCA was performed in R using TPM (transcripts per million) converted data. Differential expression was calculated using EdgeR with an initial cutoff of >0 reads in at least 1/4 of samples, and finally a cutoff of FDR <0.2 (28). For these analyses, we chose a less stringent FDR cutoff to observe greater trends in our data regarding clustering and correlated expression. All statistical calculations from edgeR are provided in Supplementary Table 1. To find differentially genes between pair and ethanol samples, LPS treatment was used as a co-variate. To find differentially expressed genes in response to LPS challenge, diet (pair and ethanol) was used as a covariate.

### Data Acquisition

We obtained miRNA expression data from M1/M2 polarized mouse bone marrow derived macrophages from previous studies (29–31). Only miRNAs with mouse and rat orthologs were considered. Represented datasets include: (1) microarray data from bone-marrow derived macrophages (BMDMs) treated with GM-BMM (M1) or M-BMM (M2) (29), (2) microarray data from BMDMs treated with IFNγ and LPS (M1) or IL-4 (M2) (30), and (3) miRNA quantitative PCR array data from BMDMs treated with IFNγ and LPS (M1), IL-4 (M2a), or IL-10 (M2c) (31). For simplicity, M2a and M2c miRNAs were combined with the other M2 miRNAs. For these datasets, only the miRNAs reported as differentially expressed were used for our analyses.

### Macrophage Polarization Enrichment

We compiled a list of 44 miRNAs as differentially expressed between polarized macrophage subtypes in mouse BMDMs from the literature (29–31). We then calculated enrichment of these miRNAs from our set of 40 differentially expressed miRNAs using the Fisher's exact test.

### miRNA Genome Clustering and Co-expression Analysis

miRNA clusters were defined as a group of 4 or more miRNAs within a 1Mb window. This definition was used to find clusters with at least 2 pri-miRNAs (−5p and −3p). For co-expression analyses, Pearson's correlation coefficients were calculated for all pair-wise comparisons of miRNAs with some expression in at least 5/6 samples (pair and ethanol at baseline). These correlation coefficients were normally distributed around 0, so we analyzed the 2.5% highest and 2.5% lowest extremes (*r* > 0.915 and *r* < −0.841, *p* < 0.05) for enrichment in miRNA clusters.

For human PBMC co-expression analyses, all qPCR data was log2 transformed, and pair-wise Pearson's correlation coefficients and *p*-values were calculated. Figures were generated using R.

### qPCR

We performed qPCR of specific miRNAs and mRNAs of interest. RNA was isolated from PBMCs and purified monocytes using the Direct-zol mini kit per the manufacturer's instructions (Zymo Research, Irvine, CA). For mRNA measurement, RNA was reverse transcribed using the Retroscript kit (Invitrogen, Carlsbad, CA) and measured with PowerSYBR qRT-PCR kits (Applied Biosystems, Foster City, CA) on aQuantStudio5 analyzer (Applied Biosystems, Foster City, CA). Relative messenger RNA (mRNA) expression was determined using gene-specific primers listed in Table 1. Analyses were performed using the ddCT method (average Ct of gene of interest–average Ct of 18S). For miRNA measurements, RNA was reverse transcribed using the miScript II RT kit (Qiagen, Hilden, Germany) with the HiSpec buffer. Relative expression was measured using the miScript SYBR Green PCR kit with miScript primers (Qiagen, Hilden, Germany) for each miRNA analyzed. Statistical analyses were performed using the ddCT method (average CT of miRNA—average CT of control SNORD68).

**Table 1:** miRNAs differentially expressed in ethanol and associated with macrophage polarization.

**GeneID**	**logFC**	**FDR**	**Polarization**	**Citations**
rno-miR-291a-5p	1.008	0.08	M2	(29, 30)
rno-miR-125a-3p	−0.65	0.07	M1	(31)
rno-miR-221-5p	−0.73	0.12	M2	(29, 30)
rno-miR-100-5p	−1.77	0	M2	(29)
rno-miR-214-3p	−1.92	0.1	M1	(31)
rno-miR-455-5p	−2.03	0.04	M1	(31)
rno-miR-152-5p	−2.04	0.01	M2	(30)
rno-miR-143-3p	−2.11	0.02	M1	(30)
rno-miR-199a-3p	−2.31	0.01	M1	(31)

### Statistical Analysis for qPCR

Data was analyzed by Analysis of Variance using a general linear model and least square means test for comparisons between groups (SAS, Carey, IN). Normal distribution of the data was assessed using the Shapiro-Wilk test and data were log-transformed, if necessary, to obtain a normal distribution. Statistical difference between groups was determined at p < 0.05, and indicated by asterisks.

## Results

### Small RNA Sequencing Reveals Dysregulation of Macrophage Polarization in Kupffer Cells From Chronic Ethanol-Fed Rats

In previous studies, expression of both M1 and M2 polarization markers in liver increased as a result of chronic ethanol feeding (12, 14, 15). We hypothesized that miRNAs might regulate polarization phenotypes in Kupffer cells isolated from ethanol-fed rats. Because no comprehensive miRNA profiles have been generated from rat immune cells, we utilized three datasets from mouse bone-marrow derived macrophages (BMDMs) stimulated *in vitro* to either an M1 or M2 phenotype to determine the miRNAs significantly associated with polarization (29–31). Each of these datasets generated M1 and M2 polarized macrophages using different stimuli, making the composite dataset highly comprehensive in detecting miRNAs associated with M1 and M2 polarization. By combining these data, we were able to generate a list of 44 miRNAs with biased expression either in M1 or M2 polarized macrophages (Supplementary Table 1). These data define a set of M1- and M2-associated miRNAs which, if upregulated or downregulated, would increase M1 or M2 polarization, respectively.

**Figure 1:**
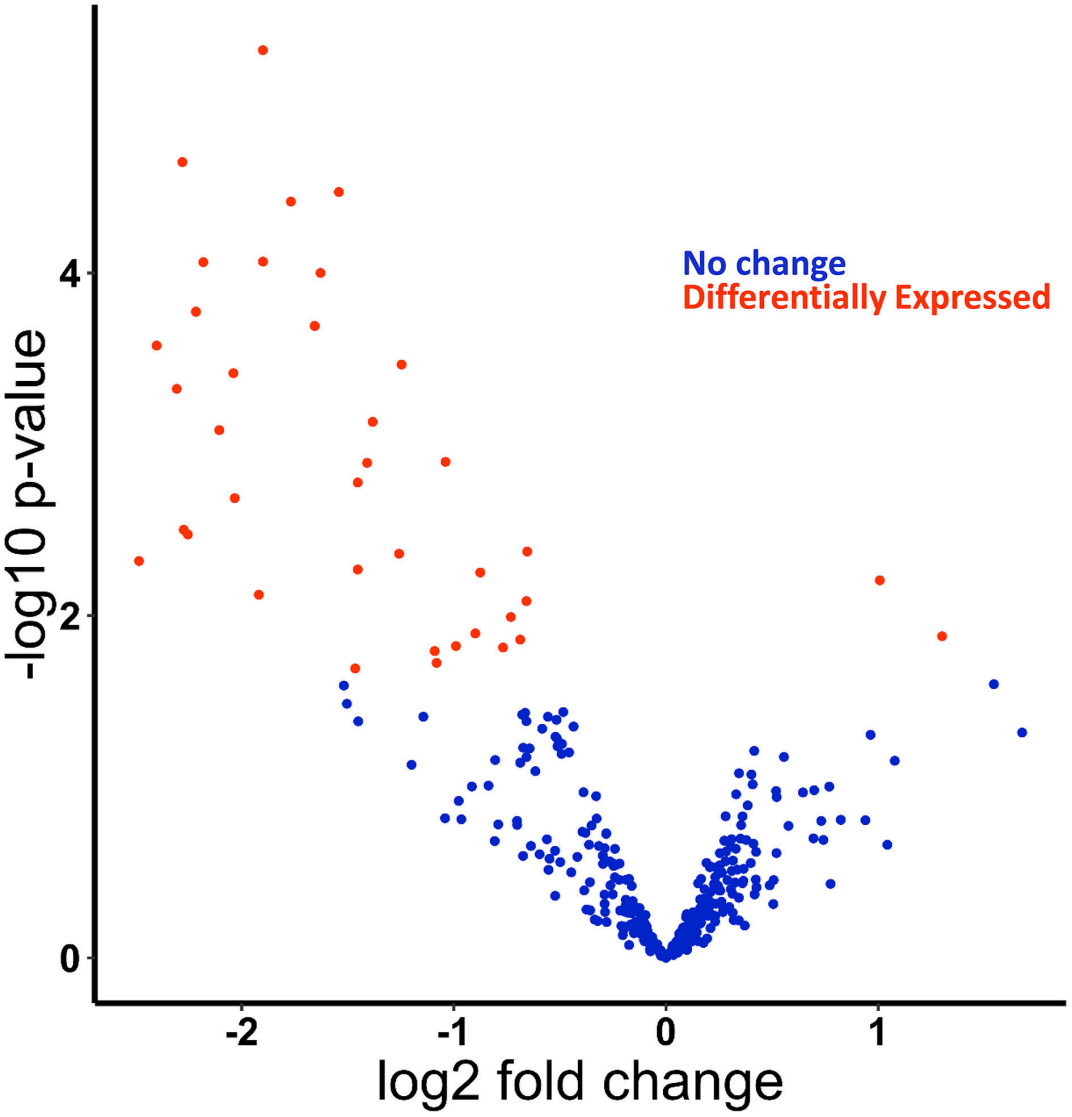
miRNAs are differentially expressed in response to ethanol feeding in primary Kupffer cells isolated from ethanol fed rats compared to pair-fed control. Small-RNA-sequencing data from Kupffer cells were analyzed for differentially expressed miRNAs. Volcano plot of differentially expressed miRNAs found using edgeR. Positive log fold change (logFC) are upregulated in Kupffer cells from ethanol-fed rats, and negative logFC are downregulated. Dots in red are statistically significant (FDR < 0.2).

We recently published small RNA-sequencing data from Kupffer cells isolated from chronic ethanol- and pair-fed rats and subsequently challenged with and without LPS (1 h) (Supplementary Figure 1) (21, 22). In our previous work, we identified and confirmed by qPCR multiple miRNAs found as up and down-regulated by ethanol in the small RNA-sequencing analysis, including miR-291b-5p and miR-181b-3p. Expression of the potential downstream targets of specific miRNAs were also investigated. For example, we identified that modulation of miR-181b-3p expression in turn regulated the expression of importin α5 (21). Following up on our previous studies using this data set, principal component analysis (PCA) of these data revealed separation of samples based on diet (Supplementary Figure 2). To assess differential expression of miRNAs, we used EdgeR to compare the effect of ethanol on miRNA expression and found in total 40 differentially expressed miRNAs: 2 upregulated and 38 downregulated in the ethanol-fed rats (Figure 1, Supplementary Figure 3, Supplementary Table 2).

Among the 44 miRNAs we identified as associated with M1/M2 polarization from published datasets (Supplementary Table 1), 9 were differentially expressed in Kupffer cells isolated from ethanol-fed rats (Table 1), showing a significant enrichment for differential expression of miRNAs associated with polarization (*p* = 0.033, Fisher's exact test). While one M2-associated miRNA (miR-291a-5p) was significantly upregulated in response to ethanol, 5 M1-associated miRNAs (miR-125a-3p, miR-214-3p, miR455-5p, miR-143-3p, and miR-199a-3p) and 3 M2-associated miRNAs (miR-221-5p, miR-100-5p, miR-152-5p) were downregulated. Differential expression of a mixture of M1 and M2 miRNAs is consistent with a hyperpolarized state.

### miRNA Expression Does Not Change in Response to Short-Term LPS Challenge in Rat Kupffer Cells

LPS challenge leads to robust expression of pro-inflammatory cytokine genes in Kupffer cells within an hour of treatment (32). Additionally, Kupffer cells isolated from ethanol-fed rats respond with exacerbated cytokine expression, due to hypersensitivity to LPS (21, 33). As a result, we predicted a similar phenotype for miRNAs. Interestingly, PCA did not show separation of LPS-treated samples in the first 10 principal components, which explained 98% of total variance, and no miRNAs were significantly differentially expressed (Supplementary Figure 4). These data indicate little to no effect of LPS on miRNA expression after 1 h of LPS challenge in Kupffer cells from either the ethanol- or pair-fed rats. We hypothesize that longer exposures are needed for LPS to modify miRNA expression in Kupffer cells. Even though the ethanol diet leads to LPS hypersensitivity in regards to inflammatory cytokines, miRNA expression dynamics appear much more stable compared to cytokine mRNAs.

### miRNAs Involved in Polarization Are Clustered Throughout the Rat Genome

**Figure 2:**
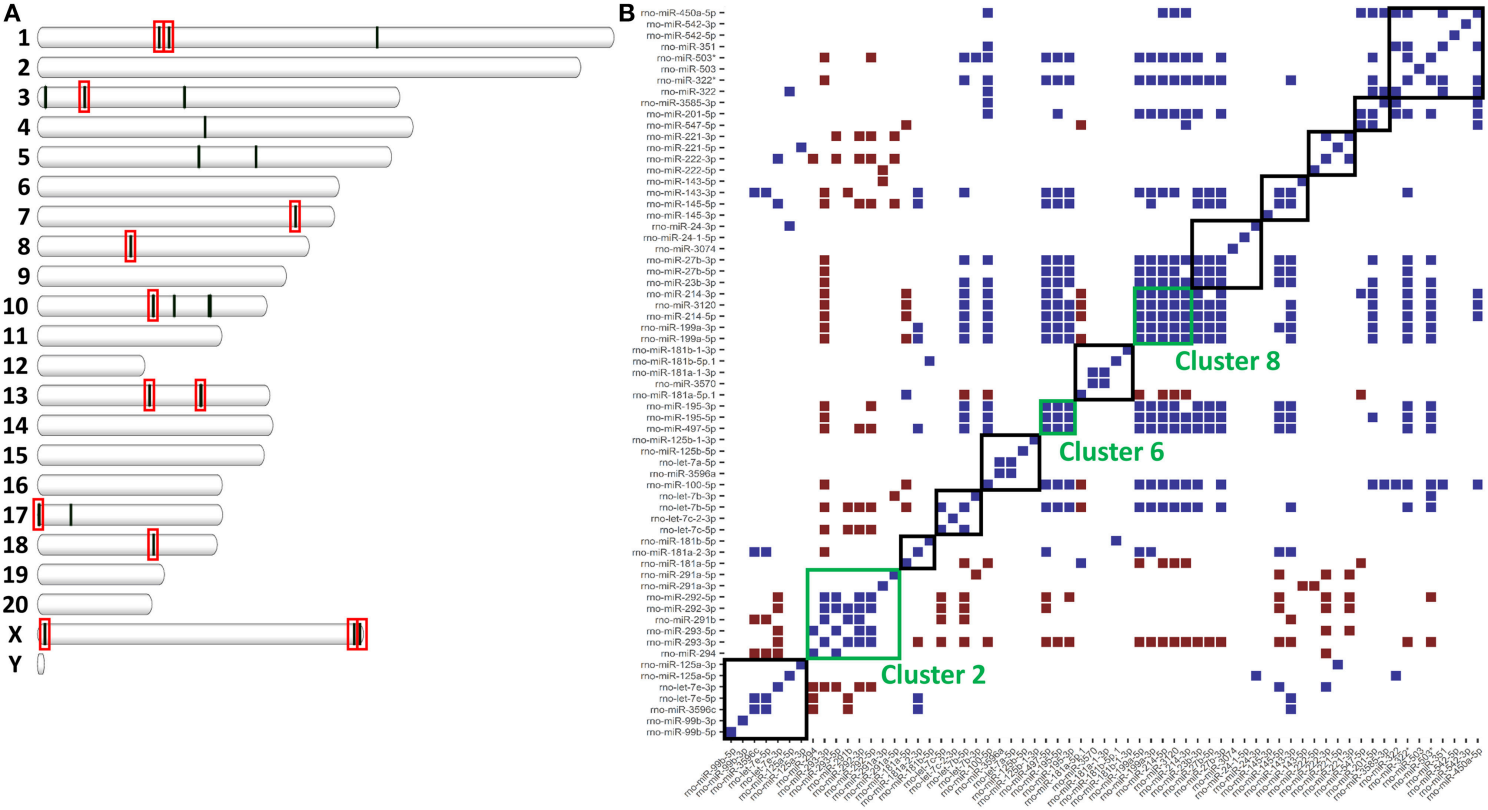
miRNAs clusters in the rat genome affect expression. **(A)** Plot of each differentially expressed miRNA in Supplementary Table 2 according to genomic location. Red boxes indicate miRNA clusters of ≥4 miRNAs. **(B)** Heatmap of significant correlations (upper and lower 2.5% of all correlations, *p* < 0.05) for all pair-wise comparisons of miRNA expression. In blue are pairs of miRNAs that are positively correlated. In red are miRNAs that are negatively correlated. Boxes indicate miRNA clusters. Green boxes are miRNA clusters significantly co-expressed (Fischer's Exact *p* < 0.05).

miRNAs are distributed throughout the genome into clusters that are either independently transcribed or transcribed and processed from the introns of host genes (19). Because miRNAs are clustered throughout the genome, we hypothesized that clustering might affect expression and allow for co-regulation (34). To assess co-regulation of closely localized miRNAs, we used the distribution of miRNAs throughout the genome to find miRNA clusters. In total, we found 13 clusters of miRNAs (defined as having 4 or more annotated miRNAs within 1Mb) that accounted for 23 of the 40 miRNAs differentially expressed in ethanol. In fact, 5 of the 13 clusters accounted for 15 of these 23 miRNAs (Figure 2A). In one of these clusters (Cluster 8), all 5 annotated miRNAs were downregulated in ethanol (miR-199a-5p, miR-199a-3p, miR-214-5p, miR-214-3p, and mir-3120) (Table 2). The remaining 8 out of 23 miRNAs were the only differentially expressed miRNA found in their cluster (Table 2). Furthermore, these 8 clusters contained additional M1 and M2 miRNAs, for example, while only miR-125a-3p was found differentially expressed, miR-125a-5p and miR-99b-3p (29–31) within the miR-125a/Let-7e/miR-99b cluster are also involved in polarization (Supplementary Table 3).

**Table 2:** miRNA clusters and their potential role in macrophage polarization.

**Cluster**	**Chr#**	**First**	**Last**	**Dir**	**Polarization**	**DE**	**Host Gene**	**Citations**
Cluster1	1	59704222	59704900	+	M1	Down	Spaca6	(31)
	rno-miR-99b-5p,rno-miR-99b-3p,rno-miR-3596c,rno-let-7e-5p,rno-let-7e-3p,rno-miR-125a-5p,**rno-miR-125a-3p**							
Cluster2	1	64536787	64538773	–	M2	Up	Nlrp12<	(29, 30)
	rno-miR-294,rno-miR-293-3p,rno-miR-293-5p,rno-miR-291b,rno-miR-292-3p,rno-miR-292-5p,rno-miR-291a-3p,**rno-miR-291a-5p**,rno-miR-290							
Cluster3	3	23150390	23151528	+	–	Down	Nr6a1<	
	rno-miR-181a-5p,**rno-miR-181a-2-3p**,rno-miR-181b-5p,rno-miR-181b-2-3p							
Cluster4	7	126590225	126590708	+	–	Down		
	rno-let-7c-5p,rno-let-7c-2-3p,rno-let-7b-5p,**rno-let-7b-3p**							
Cluster5	8	45746960	45798335	+	M2	Down	Lnc215	(29)
	**rno-miR-100-5p**,rno-miR-100-3p,rno-miR-3596a,rno-let-7a-5p,rno-let-7a-2-3p,rno-miR-125b-5p,rno-miR-125b-1-3p							
Cluster6	10	56844978	56845375	+	–	Down	RGD1308134<	
	**rno-miR-497-5p**,rno-miR-497-3p,**rno-miR-195-5p,rno-miR-195-3p**							
Cluster7	13	54952756	54952998	+	–	Down		
	rno-miR-181a-5p,rno-miR-3570,rno-miR-181a-1-3p,rno-miR-181b-5p,**rno-miR-181b-1-3p**							
Cluster8	13	80125517	80130995	+	M1	Down	Dnm3<	(31)
	**rno-miR-199a-5p,rno-miR-199a-3p,rno-miR-214-5p,rno-miR-3120,rno-miR-214-3p**							
Cluster9	17	823242	824032	+	–	Down	Npepo	
	rno-miR-23b-5p,**rno-miR-23b-3p,rno-miR-27b-5p,rno-miR-27b-3p**,rno-miR-3074,rno-miR-24-1-5p,rno-miR-24-3p							
Cluster10	18	56969921	56971352	–	M2	Down		(30)
	rno-miR-145-3p,**rno-miR-145-5p,rno-miR-143-3p**,rno-miR-143-5p							
Cluster11	X	3683940	3684566	+	M2	Down		(29, 30)
	rno-miR-222-5p,rno-miR-222-3p,**rno-miR-221-5p,rno-miR-221-3p**							
Cluster12	X	155317221	155344260	+	–	Down		
	rno-miR-509-5p, rno-miR-509-3p, rno-miR-547-5p, rno-miR-547-3p, rno-miR-201-5p, rno-miR-201-3p, rno-miR-3585-5p, **rno-miR-3585-3p**							
Cluster13	X	158148183	158153448	+	–	Down		
	rno-miR-322,rno-miR-322^*^,rno-miR-503,rno-miR-503^*^,rno-miR-351,rno-miR-351^*^,rno-miR-542-5p,**rno-miR-542-3p**,rno-miR-450a-5p,rno-miR-450a-3p							

*First and Last–chromosomal positions of entire miRNA cluster, Dir –Transcriptional direction of miRNA cluster, DE –Differential expression. Bold: significantly differentially expressed. <: Host gene in opposite direction. -: decreased in M1 and M2 in human*.

Within an individual miRNA cluster, we observed a large number of miRNAs that trended to being differentially expressed, but missed statistical cutoffs. We hypothesized that miRNAs found in clusters, if they are co-regulated, might have correlated expression across all samples. To do this, we calculated pair-wise correlation coefficients for all miRNAs in our small RNA-sequencing dataset and calculated the number of statistically significant correlation coefficients found within miRNA clusters. miRNAs in 3/13 clusters (clusters 2, 6, and 8) were significantly correlated by expression (Figure 2B), including high correlation between −5p and −3p miRNAs. −5p and −3p miRNAs originate from the same pri-miRNA, but often do not correlate well in other systems (35–37). Interestingly, these three miRNA clusters do not originate from host genes, but instead are associated with an antisense non-coding RNA. Finally, Cluster 2, the only cluster containing a miRNA significantly upregulated in response to ethanol (miR-291a-5p), was significantly anti-correlated with some of the other clusters (clusters 10 and 11).

### PBMCs From AH Patients Are Hyperpolarized

**Figure 3:**
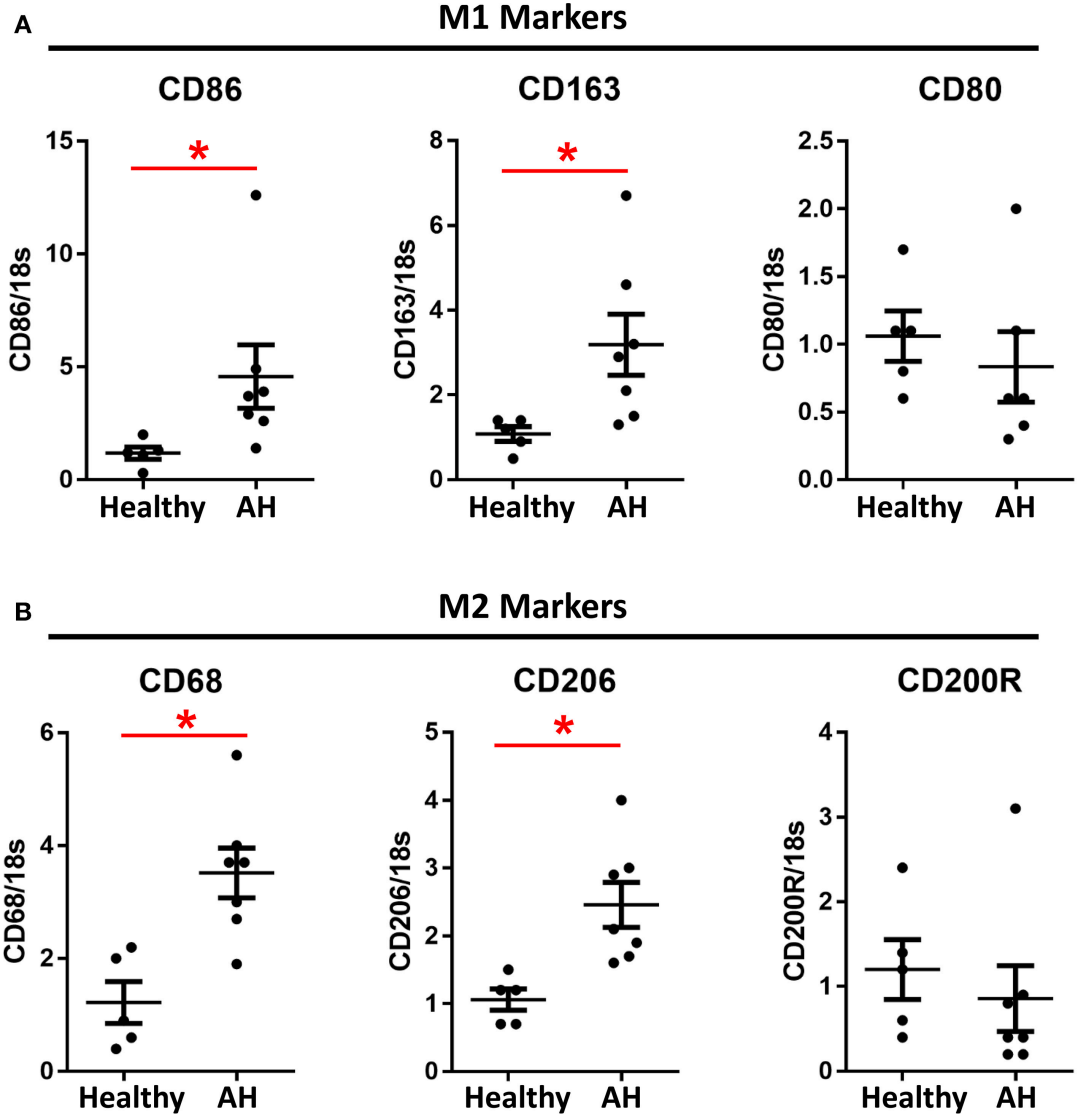
M1 and M2 polarization markers are upregulated in AH PBMCs. Expression of mRNA from PBMCs isolated from AH patients (*n* = 7) and healthy controls (*n* = 5) measured by qPCR. Values represent means ± SEM, with ^*^were significantly different from healthy controls (*p* < 0.05) **(A)** M1 polarization markers. **(B)** M2 polarization markers.

PBMCs from AH patients have been previously shown to have increased sensitivity to LPS (8, 22). Additionally, patients with alcohol use disorder have increased M1/M2 polarization after acute alcohol consumption (16, 17). PBMCs were isolated from AH patients admitted to the clinic with a median MELD score of 25.5 (IQR 23.25–33.25), ALT/AST of 37/92, and bilirubin of 19.0 (Supplementary Table 4). PBMCs from these patients had significantly higher basal expression of M1 markers, CD86 and CD163 (Figure 3A), as well as M2 markers, CD68 and CD206 (Figure 3B). However, some polarization markers, such as CD80 and CD200R, were not differentially expressed as compared to healthy controls (Figure 3).

### miRNAs Involved in Polarization Are Upregulated in PBMCs From AH Patients

**Figure 4:**
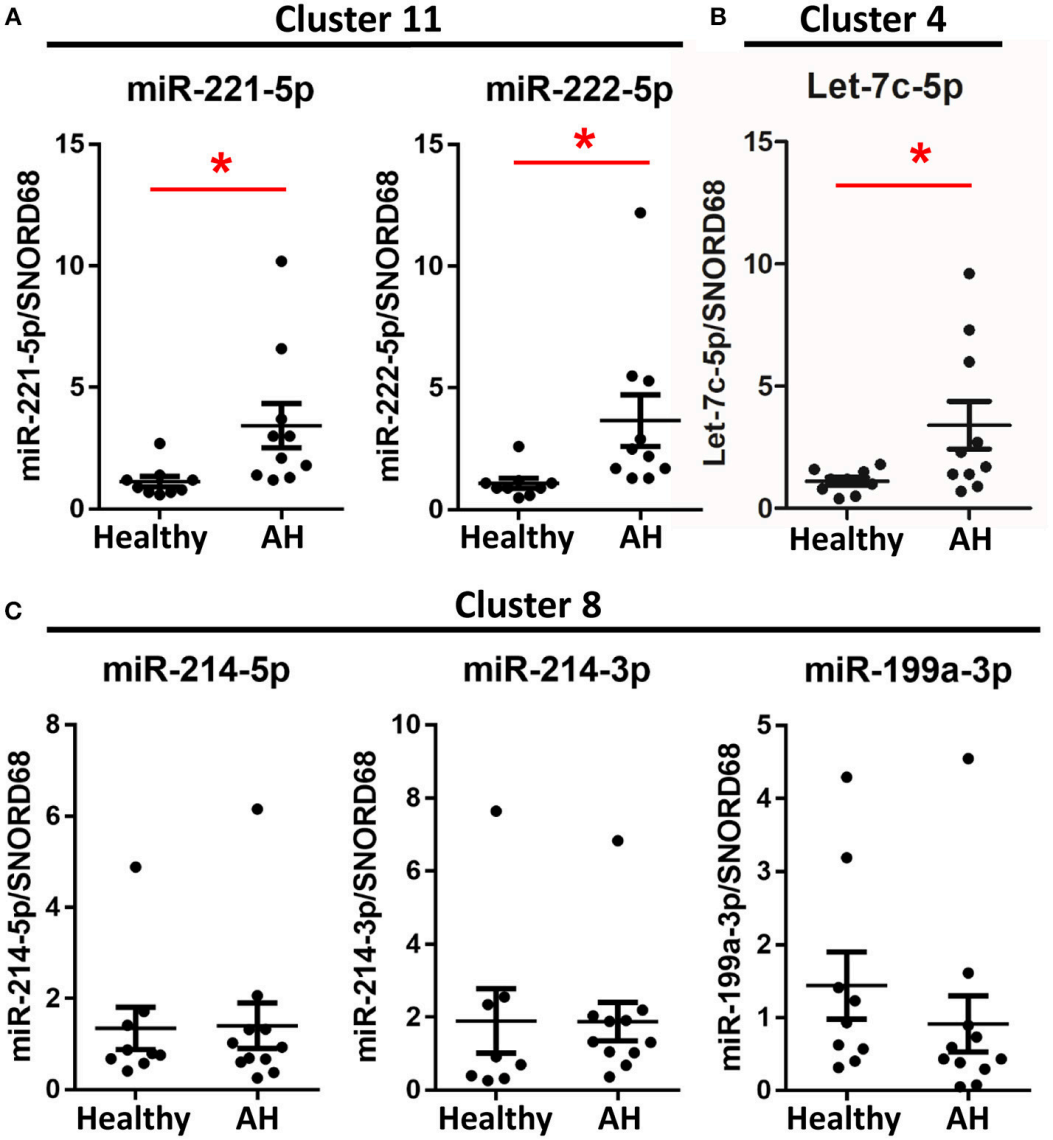
Some polarization associated miRNAs are differentially expressed in AH PBMCs. Expression of miRNA from PBMCs isolated from AH patients (*n* = 10–11) and healthy controls (*n* = 9) measured by qPCR. Values represent means ± SEM, with ^*^were significantly different from healthy controls (*p* < 0.05). Figures are organized based on clusters from Table 3. **(A)** Cluster 11, **(B)** Cluster 4, **(C)** Cluster 8.

**Figure 5:**
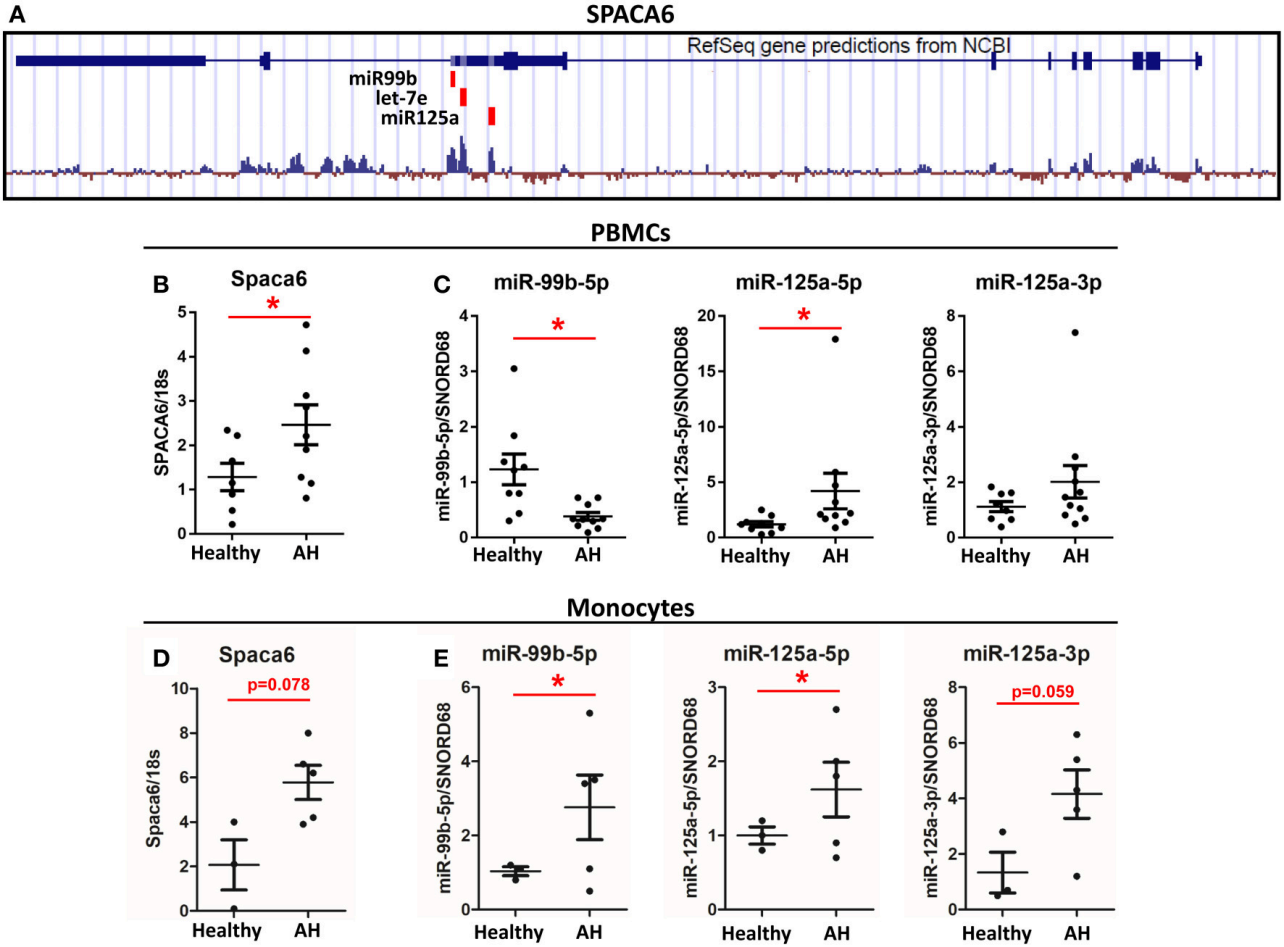
Spaca6 and miR-125a/let-7e/miR-99b cluster are differentially expressed in AH PBMCs. Analysis of multiple RNAs that originate from a single gene. **(A)** Schematic of the Spaca6 locus with miRNAs annotated. **(B,C)** Expression of mRNA and miRNAs from PBMCs isolated from AH patients (*n* = 10–11) and healthy controls (*n* = 9) measured by qPCR. **(B)** Spaca6, the host gene, expression. **(C)** miRNA expression. **(D,E)** Expression of mRNA and miRNA from isolated CD14^+^ and CD16^+^ monocytes from AH patients (*n* = 5) and healthy controls (*n* = 3) measured by qPCR. Values represent means ± SEM, with ^*^were significantly different from healthy controls (*p* < 0.05) **(D)** Spaca6, the host gene, expression. **(E)** miRNA expression. Values represent means ± SEM, with ^*^were significantly different from healthy controls (*p* < 0.05).

If PBMCs from AH patients are hyperpolarized, then we expect miRNAs associated with polarization to be similarly differentially expressed. To assess the role of miRNAs in hyperpolarization, we measured expression levels of candidate polarization miRNAs using qPCR. These include miRNAs differentially expressed in the rat Kupffer cells and other polarization-associated miRNAs. M1- (miR-221-5p and miR-125a-5p) (Figures 4A, 5C) and M2-associated miRNAs (let-7c-5p and miR-222-5p) (Figures 4B,C) were upregulated in PBMCs from AH patients, while M2-associated miRNA, miR-99b-5p, was downregulated in AH (Figure 5C). Finally, M1-associated miRNAs, miR-214-3p and miR-199a-3p, were not differentially expressed (Figure 4C). Thus, while PBMCs from AH patients are hyperpolarized compared to healthy controls, the contribution of specific M1- and M2- associated miRNAs differs compared to the ethanol-fed rat Kupffer cells.

### miRNA Clusters, but Not all Host Genes, Are Conserved Between Humans, Rats, and Mice

Among the miRNA clusters in our study, all but two (cluster 4 and cluster 12) are highly conserved between rats, mice and humans (Table 3). We hypothesized that some of the trends we observed in rats could be translated to humans. Similar to rats, miR-221/miR-222 (miRNA cluster 11), miR-125a/Let-7e/miR-99b (miRNA cluster 1), and miR-214/miR-199a (miRNA cluster 8) are clustered together in the human genome (Table 3). In humans, of Let-7c is clustered with miR-99a (cluster 4), while in rats and mice there are two copies of Let-7c: the Let-7c/miR-99a cluster and the Let-7c/Let-7b cluster. Unlike rodents, in humans each of these clusters are transcribed from dedicated non-coding RNA host genes, MiR222HG for miR-221/miR-222 and MiR99aHG for Let-7c/miR-99a, respectively (Table 3). The miR-214/miR-199a cluster is within a long non-coding RNA host gene; however in humans that host gene is antisense to an intron within DNM3 (Table 3).

**Table 3:** miRNA clusters in rat, mouse, and human.

	**Rat**		**Mouse**		**Human**	
**Cluster**	**Host Gene**	**Chr**	**Host Gene**	**Chr**	**Host Gene**	**Chr**
Cluster1	Spaca6	1	Spaca6	17	Spaca6	19
	rno-miR-99b-5p,rno-miR-99b-3p,rno-miR-3596c,rno-let-7e-5p,rno-let-7e-3p,rno-miR-125a-5p,**rno-miR-125a-3p**					
Cluster2	Nlrp12<	1	Nlrp12<	7	Nlrp12	19
	rno-miR-294,rno-miR-293-3p,rno-miR-293-5p,rno-miR-291b,rno-miR-292-3p,rno-miR-292-5p,rno-miR-291a-3p,**rno-miR-291a-5p**,rno-miR-290					
Cluster3	Nr6a1<	3	Nr6A1os	2	MiR181A2HG	9
	rno-miR-181a-5p,**rno-miR-181a-2-3p**,rno-miR-181b-5p,rno-miR-181b-2-3p					
Cluster4	unknown	7	Lncppara	15	-	-
	rno-let-7c-5p,rno-let-7c-2-3p,rno-let-7b-5p,**rno-let-7b-3p**					
Cluster4	unknown	11	Mir99aHG	26	MiR99aHG	21
	rno-miR-99a-5p, rno-miR-99a-3p, rno-let-7c-1-3p					
Cluster5	Lnc215	8	3110039I08Rik	9	miR100HG	11
	**rno-miR-100-5p**,rno-miR-100-3p,rno-miR-3596a,rno-let-7a-5p,rno-let-7a-2-3p,rno-miR-125b-5p,rno-miR-125b-1-3p					
Cluster6	RGD1308134<	10	unknown	11	miR497HG	17
	**rno-miR-497-5p**,rno-miR-497-3p,**rno-miR-195-5p,rno-miR-195-3p**					
Cluster7	unknown	13	unknown	1	miR181A1HG	1
	rno-miR-181a-5p,rno-miR-3570,rno-miR-181a-1-3p,rno-miR-181b-5p,**rno-miR-181b-1-3p**					
Cluster8	Dnm3<	13	Dnm3<	1	NR_103486.1/ Dnm3<	1
	**rno-miR-199a-5p,rno-miR-199a-3p,rno-miR-214-5p,rno-miR-3120,rno-miR-214-3p**					
Cluster9	Npepo	17	2010111I01Rik	13	C9orf3	9
	rno-miR-23b-5p,**rno-miR-23b-3p,rno-miR-27b-5p,rno-miR-27b-3p**,rno-miR-3074,rno-miR-24-1-5p,rno-miR-24-3p					
Cluster10	unknown	18	CARMN	18	CARMN	5
	rno-miR-145-3p,**rno-miR-145-5p,rno-miR-143-3p**,rno-miR-143-5p					
Cluster11	unknown	X	unknown	X	miR222HG	X
	rno-miR-222-5p,rno-miR-222-3p,**rno-miR-221-5p,rno-miR-221-3p**					
Cluster12	unknown	X	unknown	X	LOC105373347<^*^	X
	rno-miR-509-5p, rno-miR-509-3p, rno-miR-547-5p, rno-miR-547-3p, rno-miR-201-5p, rno-miR-201-3p, rno-miR-3585-5p, **rno-miR-3585-3p**					
Cluster13	unknown	X	RikencDNA	X	miR503HG	X
	rno-miR-322,rno-miR-322^*^,rno-miR-503,rno-miR-503^*^,rno-miR-351,rno-miR-351^*^,rno-miR-542-5p,**rno-miR-542-3p**,rno-miR-450a-5p,rno-miR-450a-3p					

Bold: significantly differentially expressed in rat, <: Host gene in opposite direction, ^*^: Only miR-5-9 is found in human, the rest and mouse and rat specific.

To further interrogate one of these host gene/miRNA clusters, we focused on miR-125a/miR-99b/let-7e (miRNA Cluster 1), which is within the 5′ UTR intron of the host gene Spaca6, similar to rodents (Figure 5A). Because miR-125a expression depends on Spaca6 expression, we measured basal Spaca6 mRNA in PBMCs from AH patients using qPCR. We found that Spaca6 mRNA was upregulated in human PBMCs similar to the increase in expression of miR-125a-5p, consistent with the model that these miRNAs require host gene expression (Figure 5B). Interestingly though, the other miRNAs found within Spaca6, miR-99b-5p and miR-125a-3p, were not significantly upregulated in AH. Indeed, miR-99b-5p was downregulated in AH. We predict that accumulation of miR-99b-5p is further regulated downstream of transcription (Figure 5C).

### Isolated Monocytes Have Increased Expression of Spaca6 and Associated miRNAs

CD14+ and CD16+ monocytes were isolated from the PBMCs by negative selection to see if expression of polarization-associated miRNAs differ in purified monocytes vs. the mixed population of PBMCs. As with PBMCs, the host gene Spaca6 was upregulated in the isolated monocytes from AH patients (Figure 5D). Looking specifically at the miRNAs from the Spaca6 cluster, expression of miR-125a-5p and miR-125a-3p was increased in the isolated monocytes from AH patients (Figure 5E). Interestingly, expression of miR-99b-5p increased in the AH monocytes, which is in contrast to the response in the PBMCs (Figure 5E). These results are consistent with our model that intronic miRNAs require host gene expression, and that these polarization-associate miRNAs are dysregulated in AH monocytes. The difference between isolated monocytes and PBMCs is likely due to differential regulation in the expression of these miRNAs in other cell types present in the mixed immune cells populated in PBMC preparations, for example miR-125a and miR-99b are also expressed in CD4^+^ and CD8^+^ T-cells (38).

### miRNA Clusters Are Coordinately Expressed

Because host genes regulate miRNA expression, we predicted that miRNAs within the same host gene would have highly correlated expression. Among the miRNAs differentially expressed in AH, miRNAs from the same cluster behaved similarly; miR-221-5p and miR-222-5p miRNAs (within miRNA cluster 11) were both upregulated in AH, while miR-214-5p, miR-214-3p, and miR-199a-3p (miRNA cluster 8) were not (Figures 4A,C).

**Figure 6:**
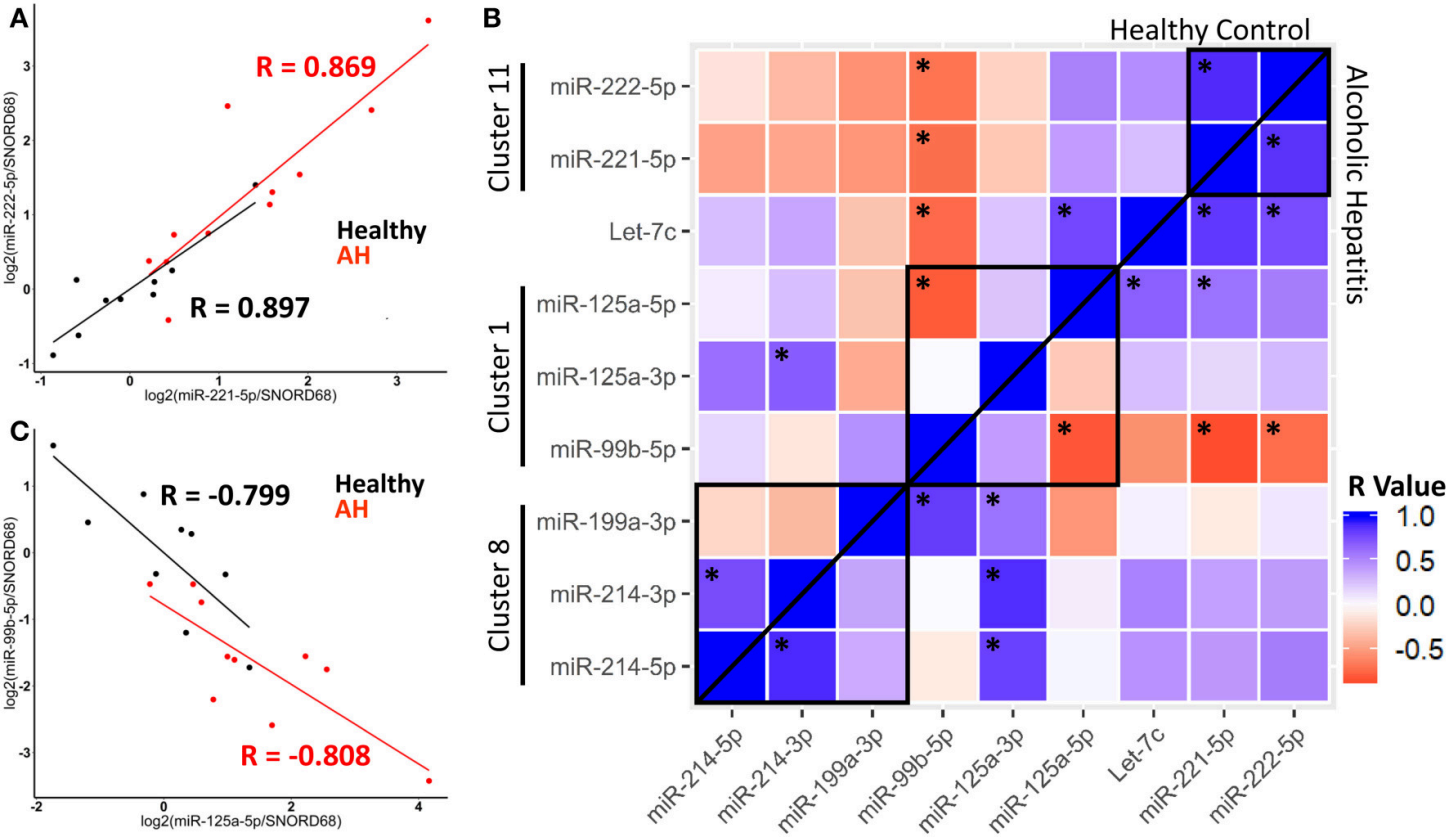
miRNA expression within clusters are correlated in healthy controls and AH patients. **(A,C)** Scatterplot of log2 transformed miRNA expression data from qPCR measurements. Black–Healthy Control, Red–AH patient. R values are Pearson's correlation coefficients, (*p* < 0.05). **(B)** Heatmap of correlations for all pair-wise comparisons of miRNA expression, with ^*^representing significant correlation (*p* < 0.05). Black boxes are miRNA clusters.

We observed correlation in expression between clustered miRNAs, for example, miR-221-5p and miR-222-5p were highly co-expressed in healthy controls and AH patients (Figure 6A). An important note, expression of miR-221-5p and miR-222-3p was also highly correlated in the rat Kupffer cells, although miR-221-3p and miR-222-3p were not (Figure 2B).

We calculated pair-wise correlation coefficients for all miRNAs measured by qPCR. Expression of many miRNAs within clusters, and even some miRNA outside of clusters were significantly correlated, possibly due to their common role in macrophage polarization. Interestingly, correlated expression of miRNAs was observed in both healthy controls and AH patients, suggesting that AH modulates the accumulation of miRNAs, but not their coordinated expression within a cluster (Figure 6B, above and below line). For example, the miR-125a/miR-99b/let-7e cluster (within Spaca6), was significantly anti-correlated between miR-125a-5p and miR-99b-5p in both healthy controls and AH patients (Figure 6C). These data indicate that while miRNA clusters and host genes regulate expression, miRNA maturation and downstream degradation also likely plays a role in regulating the accumulation of miRNAs.

## Discussion

Alcoholic Hepatitis is characterized by macrophage hypersensitivity to innate immune stimuli. In this study, we sought to understand the regulation of M1 and M2 hyperpolarization in macrophages in AH using two models for innate immune dysfunction as a result of chronic alcohol consumption: primary culture of Kupffer cells isolated from rats fed chronic ethanol and PBMCs isolated from AH patients. In both cell types, alcohol increased the expression of mRNAs and miRNAs characteristic of diverse M1 and M2 polarized cells. Many of these miRNAs were found in clusters throughout the genome; some of these clustered miRNAs had highly correlated expression, indicating possible co-regulation. These miRNA clusters are found within host genes; mRNAs that are transcribed and spliced in order to generate mature miRNAs. Interestingly, host gene expression did not correlate with co-regulation of miRNA clusters while the presence of antisense non-coding RNAs were more predictive of cluster co-regulation.

miRNAs play an important role in macrophage polarization. Studies focused on mouse BMDMs and human PBMCs have implicated a diverse array of miRNAs differentially expressed in all polarization subtypes. For our work, we compiled miRNA datasets from mouse to discover new roles for macrophage regulation in response to alcohol. Using our previously published miRNA sequencing data from Kupffer cells isolated from ethanol and pair-fed rats, we discovered enrichment of miRNAs associated with both M1 and M2 polarized macrophages, indicative of a more complex M1/M2 polarization phenotype that can be described as hyperpolarization. Moreover, expression of M1 and M2 associated cell surface markers and miRNAs were increased in PBMCs isolated from AH patients compared to healthy controls. Interestingly, while alcohol leads to hyperpolarization in both rat Kupffer cells and PBMCs from AH patients, the miRNAs associated with hyperpolarization differed between the two models. As a result, hyperpolarization is likely very different between the rat Kupffer cells and the PBMC; the different miRNAs will significantly impact protein expression and phenotypes in respective cells. Currently, we cannot determine if these differences are due to differing species, cell type or disease phenotype.

To understand how these differentially expressed miRNAs are regulated, we explored different mechanisms of how they might be transcribed. miRNAs are predominantly transcribed from the genome via two ways: as independent transcripts or co-transcribed within host genes. Additionally, subsets of miRNAs are clustered together, either as genes with very close proximity, or the result of sharing a single host gene. In both rat Kupffer cells and human PBMCs, many of the differentially expressed miRNAs were localized to clusters. These clusters are interesting because, if they are co-regulated, then changing expression of a cluster of miRNAs would likely amplify the signal, having a much greater impact on protein expression. In fact, many clustered miRNAs are well-conserved across species, though many of the regulating host genes have not been fully characterized or annotated. Additionally, evidence is accumulating that miRNA clusters function in overlapping networks (34, 39–41). For example, the most well-studied miRNA cluster, the miR-17-92 cluster, targets numerous overlapping mRNAs including genes involved in cell cycle progression and apoptosis (34, 42).

In rat Kupffer cells, we comprehensively characterized the relevant miRNA clusters with known roles in macrophage polarization, and in many cases, clusters housed multiple polarization associated miRNAs. We hypothesize that macrophage polarization requires the contribution of many miRNAs that target multiple signaling pathways in order to shift cellular phenotypes. Many of these polarization-associated miRNAs are found in clusters and co-regulated. This implies a robust mechanism is required to change the cellular phenotype. Future work will need to tease apart the separate contributions of each miRNA to polarization, as well as the possible differences in regulation between macrophage/monocyte subtypes, i.e., resident macrophages vs. peripheral monocytes.

Three of the identified miRNA clusters were highly co-expressed, implying functional co-regulation. Surprisingly, these clusters did not have an annotated host gene to regulate expression in the rat genome, but instead each genomic loci contained an antisense transcript. How regulation of these non-coding RNAs leads to coordinate expression of miRNAs is unknown. Specific antisense transcripts have known roles in regulating gene expression, especially of nearby targets, but most of these studies are limited to co-regulated mRNAs (43, 44). These antisense transcripts have sequence complementarity to the clustered miRNAs and would be expected to bind and repress these miRNAs with high affinity. Further work will have to be done to understand the role of antisense non-coding RNAs in miRNA expression, maturation, and possibly repression.

Interestingly, these miRNA clusters are well-conserved between rats, mice and humans, but in many cases the host genes surrounding them differ considerably, especially in the case of the rat genome which has only a few annotated host genes. The human genome has annotated host genes for almost all of these clusters, indicating either divergence in the regulation of these genes in humans compared to rodents and/or poor annotation in rats and mice. Our qPCR results from the human PBMCs indicate co-regulation among a few of these clusters, which all originate from host genes.

In the isolated monocytes, miR-125a-5p, miR-125a-3p, and miR-99b-5p were upregulated in AH patients as was its host gene Spaca6, consistent with an association between increased host gene expression and increased miRNA expression. Spaca6, or sperm acrosome associated protein 6, has not previously been implicated in alcoholic hepatitis or the immune system, even though the miRNAs clustered within Spaca6 have known roles in the immune system. We hypothesize that the primary role for Spaca6 in cells is to act as the host gene for miRNAs, thus upregulation of Spaca6 leads to upregulation of the associated miRNAs. Interestingly, the Spaca6 cluster of miRNAs (miR-125a-5p, let-7e-5p, and miR-99b-5p) are upregulated in response to prolonged LPS challenge in PBMCs. miR-125a-5p and let-7e-5p down regulate TLR signaling by targeting inflammatory cytokines and in the case of miR-125a-5p, directly inhibiting TLR4 (45). Interestingly, other groups have found that NFκB signaling and miR-125a expression are tightly linked as NFκB can upregulate miR-125a while miR-125a-5p and miR-99b-5p downregulate expression of multiple TLRs (46, 47). Additionally, IL-4 and IFNs can suppress miR-125a-5p expression (48). In alcoholic hepatitis patients, PBMCs chronically encounter low levels of LPS, possibly indicating a mechanism for the increase in miR-125a-5p and miR-99b-5p observed in AH. Further work needs to be done to understand how different signaling pathways may intersect and regulate miRNA expression and maturation. Interestingly, in the PBMCs, which contain a mixture of cell types, miR-99b-5p was downregulated in the AH patients and expression of miR-125a-5p was anti-correlated with miR-99b-5p expression, in both healthy controls and AH patients. We hypothesize that this anti-correlated expression is due to the other cell-types present in PBMCs, for example miR-125a and miR-99b are expressed in T-cells (38). How T-cells may be dysregulated in AH is unknown, but our results would suggest the possibility of other immune cells contributing significantly to disease.

We also analyzed the effect of LPS challenge on miRNAs in rat Kupffer cells and discovered little to no effect on expression after 1 h. LPS challenge has a dramatic effect on mRNA expression, such as upregulation of pro-inflammatory cytokines, with exacerbated responses due to alcohol (12, 13, 15). We hypothesize that this stability in miRNA expression is indicative of their homeostatic role in the cell; because miRNAs function to regulate large networks of genes, their expression may not change quickly in the cells. Longer and higher dose exposures to LPS may be required to see a response. Finally, these data indicate that the differences in miRNA expression in response to alcohol may be remarkably stable, and more work is required to understand how these changes can be reversed, or restore miRNA to homeostasis.

## Conclusion

miRNAs regulate diverse cellular functions by modulating the expression of proteins. In this work, we identified miRNAs that regulate macrophage/monocyte polarization, and leverage that data to understand hyperpolarization in alcoholic hepatitis, both in patients and rodent models. We also explored transcriptional regulation of miRNAs and found a number of polarization associated miRNAs were clustered throughout the genome, and number of these clusters had highly correlated expression. These data together help us to understand how dysregulated miRNAs may lead to a shift in macrophage/monocyte polarization, and hypersensitivity to microbes and microbial products in alcoholic hepatitis.

## Data Availability

The small RNA sequencing datasets for this study can be found at NCBI GEO under accession number GSE95403. All codes used for analyses can be found at github (github/atomadam2).

## Author Contributions

AK, PS, and LN contributed conception and design of the study. PS performed small RNA sequencing. AK performed PBMC studies, bioinformatics analyses, and statistical analyses. AK wrote the first draft of the manuscript. AK and LN wrote sections of the manuscript. All authors contributed to manuscript revision, read and approved the submitted version.

### Conflict of Interest Statement

The authors declare that the research was conducted in the absence of any commercial or financial relationships that could be construed as a potential conflict of interest.

## Supplementary Material

Supplementary Figure 1 Schematic of Kupffer cell isolation from rats, LPS challenge, and small RNA isolation and sequencing.

Supplementary Figure 2 PCA analyses reveal separation of Kupffer cells isolated from ethanol-fed rats (E) compared to pair-fed controls (P). PCA does not reveal separation of cells *ex vivo* challenged with LPS (blue–basal, red–LPS). Number refer to paired samples.

Supplementary Figure 3 Schematic of small RNA analysis from Kupffer cells.

Supplementary Figure 4 Volcano plot of differentially expressed miRNAs with respect to LPS treatment (FDR < 0.2).

Supplementary Table 1 List of all miRNAs with role in macrophage polarization in mouse BMDMs.

Supplementary Table 2 Results of edgeR analysis for differentially expressed miRNAs in Kupffer cells isolated from ethanol-fed rats.

Supplementary Table 3 Detailed and complied list of all miRNA clusters in rat with known roles in macrophage polarization, and differential expression in rat Kupffer cells.

Supplementary Table 4 Clinical parameters for patients from which we isolated PBMCs.
