# Supplementary material for: miRNAs Involved in M1/M2 Hyperpolarization Are Clustered and Coordinately Expressed in Alcoholic Hepatitis
Source: Front Immunol. 2019 Jun 7;10:1295. doi: 10.3389/fimmu.2019.01295 (PMC6568035; doi:10.3389/fimmu.2019.01295)
Supplement: Supplementary Figure 1 — Schematic of Kupffer cell isolation from rats, LPS challenge, and small RNA isolation and sequencing. [file Data_Sheet_1.pdf]

# miRNAs involved in M1/M2 hyperpolarization are clustered and coordinately expressed in Alcoholic Hepatitis

## Supplemental Figures

Adam Kim, Paramananda Saikia, Laura E. Nagy

Supplemental Figure 1

Experimental Design

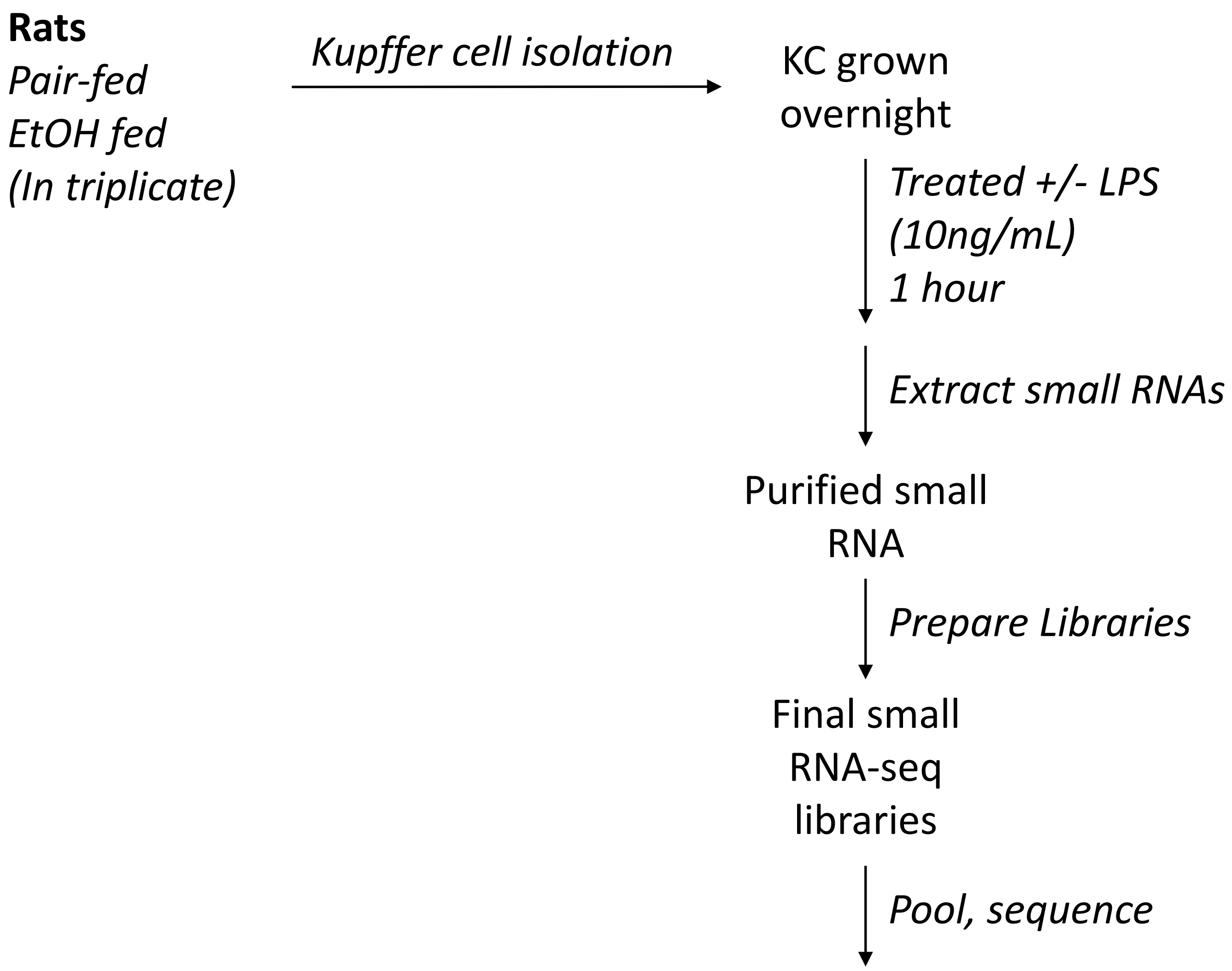


Supplementary Figure 1 - Schematic of Kupffer cell isolation from rats, LPS challenge, and small RNA isolation and sequencing.

Supplemental Figure 2

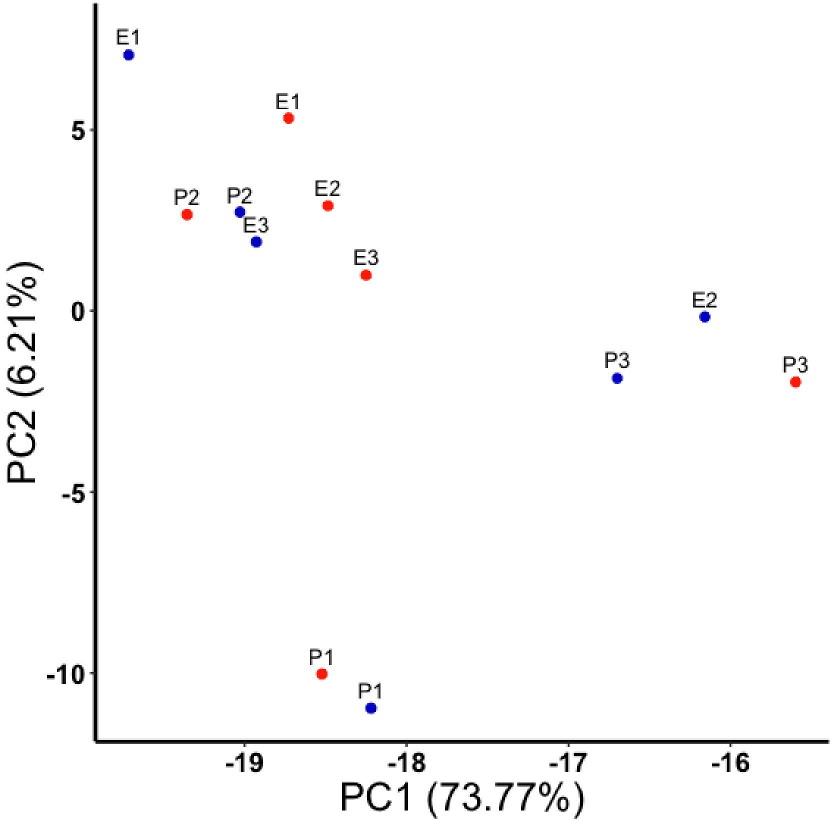


Supplementary Figure 2 PCA analyses reveal separation of Kupffer cells isolated from ethanol-fed rats (E) compared to pair-fed controls (P). PCA does not reveal separation of cells *ex vivo* challenged with LPS (blue – basal, red – LPS). Number refer to paired samples.

Supplemental Figure 3

Analysis Pipeline

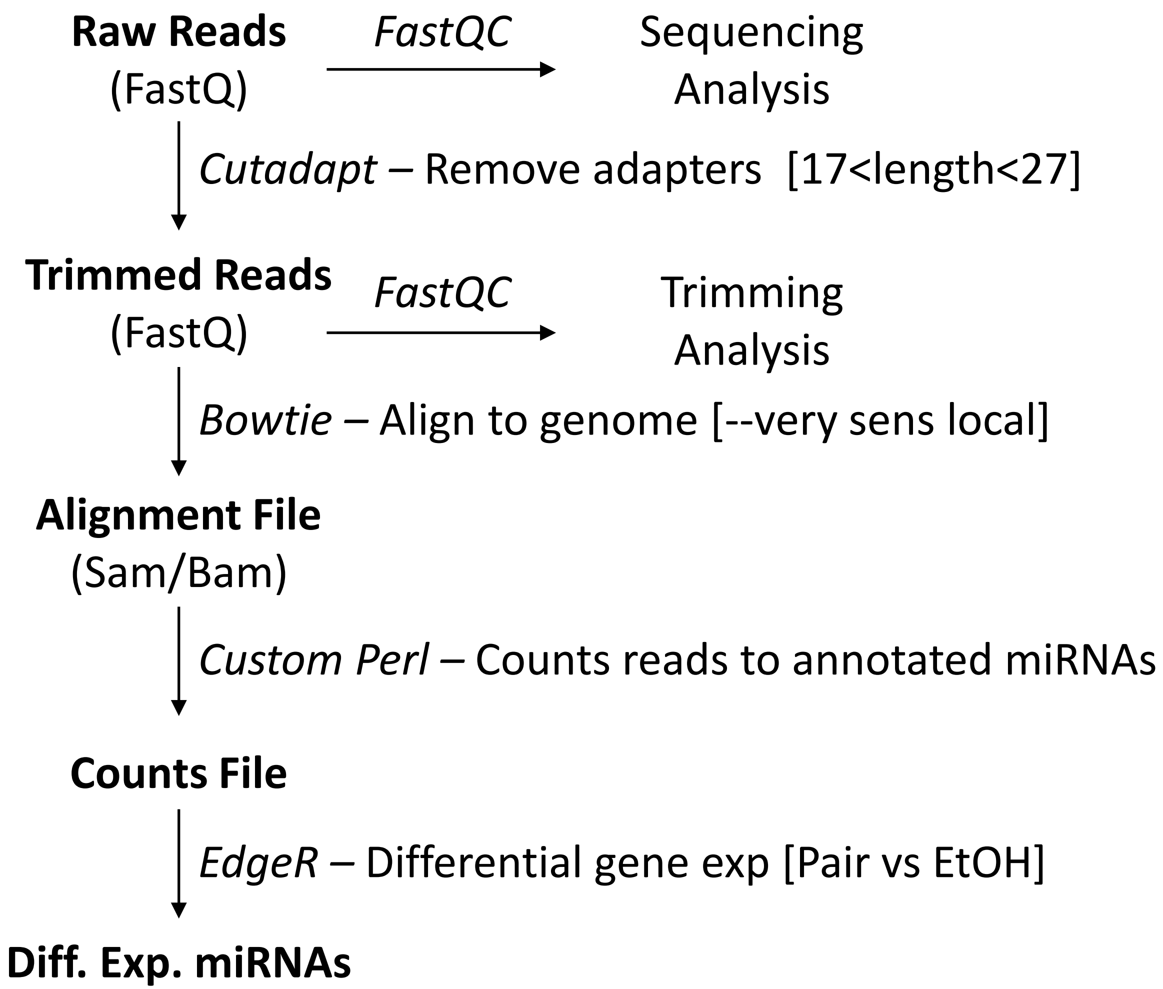


Supplementary Figure 3 - Schematic of small RNA analysis from Kupffer cells.

Supplemental Figure 4

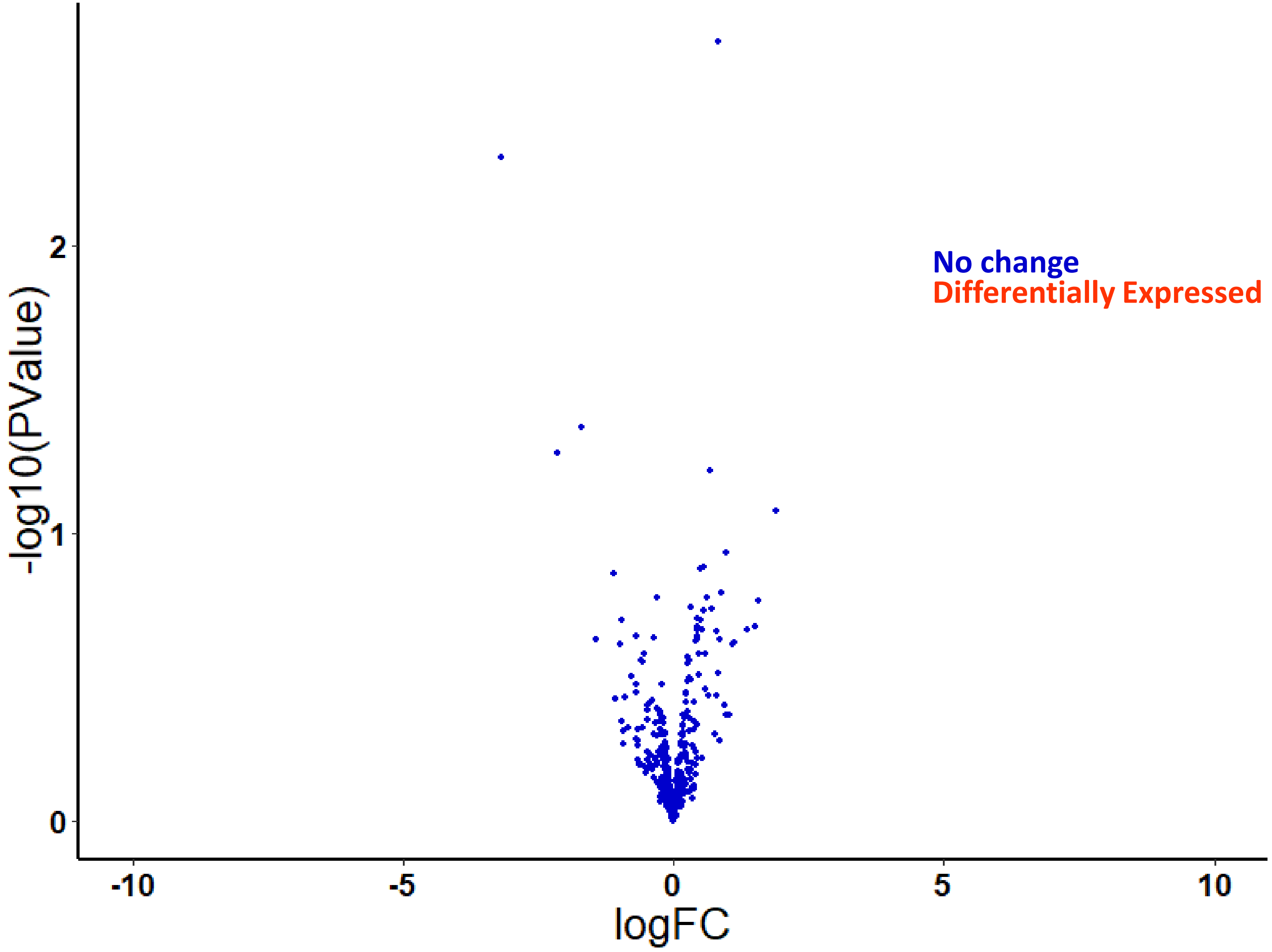


Supplementary Figure 4 - Volcano plot of differentially expressed miRNAs with respect to LPS treatment (FDR<0.2).
